# Effectiveness and retention of certolizumab pegol and JAK inhibitors in rheumatoid arthritis: a multicenter retrospective study stratified by rheumatoid factor status

**DOI:** 10.3389/fmed.2026.1865270

**Published:** 2026-09-09

**Authors:** Mitndbaim Parra-Moreno, Lourdes Ladehesa-Pineda, Jerusalem Calvo-Gutierrez, Francisco José Cepas-Guitérrez, Elena Moreno-Caño, Montserrat Robustillo-Villarino, Andrés Zúñiga-Vera, Chamaida Plasencia-Rodríguez, Ana Martinez-Feito, Ester Costa-Moya, Delia Taverner-Torrent, Luis Sainz-Comas, César Díaz-Torné, Virginia Ruiz-Esquide, Cynthia Rojas, Lourdes Martin-de la Sierra Lopez, Laura Jiménez-Rodríguez, David Velasco-Sánchez, Regina Faré-García, Antoni Juan-Mas, Marina Soledad Moreno-García, Ana Valeria Acosta-Alfaro, Santiago Muñoz-Fernández, María Martín-López, Raquel Zas, Javier Godoy-Navarrete, Isabel Añon-Oñate, Fernando Ortiz-Márquez, Natalia Mena-Vázquez, Paula Estrada, Rafaela Ortega-Castro, Alejandro Escudero-Contreras, Clementina López-Medina

**Affiliations:** 1 Rheumatology Department, Reina Sofia University Hospital, IMIBIC, University of Cordoba, Cordoba, Spain; 2 Rheumatology Department, La Plana University Hospital, Vila-Real, Spain; 3 Rheumatology Department, La Paz University Hospital, IdiPAZ, Madrid, Spain; 4 Rheumatology Department, Sant Joan Reus Hospital, Reus, Spain; 5 Rheumatology Department, Santa Creu I Sant Pau Hospital, Barcelona, Spain; 6 Rheumatology Department, Clinic Hospital, Barcelona, Spain; 7 Rheumatology Department, Ciudad Real University General Hospital, Ciudad Real, Spain; 8 Rheumatology Department, Son Llazter University Hospital, Palma, Spain; 9 Rheumatology Department, Miguel Servet University Hospital, Zaragoza, Spain; 10 Department of Rheumatology, FIIB HUIS-HUHEN, Hospital Universitario Infanta Sofía, Madrid, Spain; 11 Department of Medicine, Faculty of Medicine, Health and Sports, Universidad Europea de Madrid, Madrid, Spain; 12 Rheumatology Department, Doce de Octubre University Hospital, Madrid, Spain; 13 Rheumatology Department, Jaén University Hospital, Jaén, Spain; 14 Rheumatology Department, Regional University Hospital of Malaga, IBIMA, Malaga, Spain; 15 Rheumatology Department, Moises Broggi Hospital, Sant Joan Despí, Spain

**Keywords:** certolizumab pegol, clinical effectiveness, drug retention, JAK inhibitors, rheumatoid factor

## Abstract

**Background:**

Patients with rheumatoid arthritis (RA) and high levels of Rheumatoid Factor (RF) often present with a higher inflammatory burden and may show a reduced response to JAK inhibitors (JAKi). However, previous studies have demonstrated that certolizumab pegol (CZP) provides comparable efficacy in patients regardless of RF level.

**Objectives:**

To compare the effectiveness and treatment retention of CZP versus JAKi in patients with RA according to baseline RF levels, assessing (a) changes in disease activity at 6 and 12 months, and (b) treatment retention over a 3-year follow-up period.

**Methods:**

This multicentre retrospective cohort included RA patients initiating CZP or JAKi (baricitinib, filgotinib, tofacitinib, upadacitinib) between 2010 and 2024. Patients were stratified by baseline RF levels (RF < 189 IU/mL and RF ≥ 189 IU/mL). Disease activity was assessed using DAS28-ESR at baseline, 6, and 12 months. Drug retention was analysed using Cox regression models, with a 3-year follow-up. A sensitivity analysis using IPTW was performed to account for baseline differences, while MICE and MNAR sensitivity analysis addressed missing data and informative missingness.

**Results:**

The study included 370 RA patients (409 treatment episodes: 234 CZP, 175 JAKi). Among patients with RF ≥ 189 IU/mL, CZP was associated with a greater reduction in DAS28-ESR than JAKi at both 6 and 12 months, with statistically significant differences at 12 months. In contrast, among patients with RF < 189 IU/mL, a greater reduction in DAS28-ESR at 6 months was observed in the JAK inhibitor group. CZP also demonstrated substantially higher drug retention at 3 years. In IPTW-weighted analyses, CZP was associated with a greater improvement in disease activity at 12 months in patients with RF ≥189 IU/mL, while treatment retention was numerically higher with CZP than with JAKi in this subgroup. However, the 12-month difference in clinical efficacy did not remain statistically significant under the MNAR sensitivity analysis.

**Conclusion:**

This study suggests that CZP may be associated with greater improvement in disease activity at 12 months in patients with RA and high RF levels, although this finding did not remain statistically significant under the MNAR sensitivity analysis and should therefore be interpreted with caution. A numerical difference in treatment retention favouring CZP was also observed. These findings highlight the potential importance of considering RF levels when selecting treatment and warrant further investigation in prospective studies.

## Introduction

Rheumatoid arthritis (RA) is a chronic autoimmune disease characterized by polyarthritis, joint damage, and functional impairment. A key immunological marker associated with RA development is rheumatoid factor (RF), an autoantibody directed against the fragment crystallizable (Fc) region of IgG. This autoantibody may form immune complexes contributing to the inflammatory processes central to RA pathogenesis (1).

Elevated RF levels have been associated with greater disease severity and increased cardiovascular risk (2). In fact, high RF levels have been linked to greater radiographic progression (3) and higher disease activity (4). For these reasons, the 2022 update of the European Alliance of Associations for Rheumatology (EULAR) recommendations for the management of RA included the presence of RF (particularly at high levels) as a poor prognostic factor (5).

In this context, elevated RF levels have also been associated with a lower response and retention rate to biologic disease-modifying antirheumatic drugs (bDMARDs), particularly Tumor Necrosis Factor inhibitors (TNFi) that contain an Fc region. This may be explained by the ability of RF to bind to the Fc fragment of these drugs, leading to reduced drug levels (6). However, the absence of an Fc region in certolizumab pegol (CZP), a pegylated TNFi, may confer more favorable outcomes in patients with high RF levels. This was demonstrated in a recent retrospective study, where CZP serum levels at 6 months of follow-up were similar regardless of RF levels, whereas patients treated with adalimumab or infliximab showed lower drug levels in RF-positive patients compared to seronegative ones (7). Subsequent analyses supported this hypothesis, confirming that in patients with high RF levels, treatment response and drug retention were lower for Fc-containing TNFi compared to CZP (8, 9).

Recently, Janus kinase inhibitors (JAKi) have been approved for the treatment of RA. These agents belong to the class of targeted synthetic DMARDs (tsDMARDs) and act by blocking Janus kinases (enzymes involved in the signaling pathways of various pro-inflammatory cytokines) thereby reducing inflammation, immune activation, and disease symptoms. Unlike bDMARDs, JAKi are small-molecule, oral agents that act intracellularly.

Given the emerging evidence that the Fc portion of TNF inhibitors may negatively influence drugs levels and clinical outcomes in patients with high RF levels, CZP has gained particular interest as a potentially more effective option in this population. Several ongoing studies are currently comparing CZP with other Fc-containing or Fc-independent bDMARDs. However, no previous research has evaluated whether JAK inhibitors (another Fc-independent therapeutic class) perform differently from CZP in patients with high RF levels. Since JAK inhibitors act intracellularly and do not contain an Fc region, they would theoretically be unaffected by RF; yet, high RF levels may still indicate a greater inflammatory burden, potentially influencing treatment response. These considerations provide the rationale for comparing CZP and JAK inhibitors specifically in RA patients with high RF levels.

For these reasons, we conducted this study to compare (a) the effectiveness of CZP versus JAKi at 6 and 12 months of treatment, and (b) the drug retention rate between both groups over a 3-year follow-up period according to baseline RF levels.

## Methods

### Study design and population

This is a longitudinal, retrospective, multicentre study conducted across fourteen rheumatology centres in Spain. The study included patients diagnosed with RA by their treating rheumatologist, based on the 2010 American College of Rheumatology (ACR) classification criteria (10). Eligible patients were those who received treatment with CZP or JAKi (baricitinib, filgotinib, tofacitinib and upadacitinib) between 2010 and 2024, in any line of therapy. A patient could be included multiple times if they received more than one treatment episode in this study.

The study was approved by the Ethics Research Committee of Córdoba (Spain) on 30 July 2024, under the code SICEIA-2024-001712. The Committee granted a waiver of informed consent, as all data were collected from clinical records and did not influence prescription decisions or involve any intervention with the patients.

### Variables

Several variables were collected from patients' medical records at the time of treatment initiation, including sociodemographic characteristics (sex, age, and current smoking status), disease duration (time between symptom onset and treatment initiation), and concomitant use of methotrexate. The treatment line of CZP or JAKi initiation (i.e., whether it was used as a first- or later-line therapy) was also recorded.

Information from routine blood samples was also collected at the time of treatment initiation. Erythrocyte sedimentation rate (ESR), anti-citrullinated protein antibodies (ACPA) and/or RF positivity was recorded, along with the RF level. For this analysis, a baseline RF level ≥189 IU/mL was considered “high RF”, based on the value of the fourth quartile in this population (8, 9).

Disease activity was measured using the Disease Activity Score (DAS) using 28 joint counts (DAS28) (11) at baseline, 6 and 12 months of follow-up. DAS28-ESR was considered, as erythrocyte sedimentation rate (ESR) is generally considered less affected by the direct pharmacodynamic effects of JAK inhibitors on C-reactive protein (CRP), which can occur independently of clinical improvement.

For patients who contributed more than one treatment to the analysis, baseline data were recorded at each treatment initiation.

### Statistical analysis

Descriptive statistics are presented as means and standard deviations (SD) for continuous variables, and as absolute frequencies and percentages for categorical variables.

Baseline characteristics between the CZP and JAKi groups were compared using the chi-square or Fisher's exact test for categorical variables, and the Student's t-test or Mann–Whitney U test for continuous variables, depending on data normality.

Patients receiving CZP or JAKi were stratified into two groups based on baseline RF levels: ≥189 IU/mL and <189 IU/mL. To compare treatment response between CZP and JAKi, the change in DAS28 from baseline to month 6 and from baseline to month 12 was calculated and compared within each RF stratum (≥189 IU/mL and <189 IU/mL), to assess whether treatment response differed by baseline RF level.

To evaluate drug retention, Kaplan–Meier survival curves and log-rank tests were used in the overall population and according to baseline RF levels. For survival analyses, follow-up was truncated at 3 years, as JAKi were introduced later than CZP and longer retention times for CZP could be influenced by regulatory approval dates. Cox proportional hazards models were also performed, with CZP as the reference group. Since some patients contributed more than one treatment episode, each treatment episode was analysed as a separate observation, with survival time calculated from the start of that treatment episode.

Treatment discontinuation for any reason was considered an event. Treatment episodes without discontinuation were censored at the earliest of three years after treatment initiation, the last documented follow-up while the patient was still receiving treatment, or the end of the study period.

As a sensitivity analysis, treatment effects and retention rate were evaluated using inverse probability of treatment weighting (IPTW) (12), with all analyses stratified a priori according to RF levels (<189 vs. ≥189). Within each RF stratum, propensity scores were estimated using multivariable logistic regression including sex, age, disease duration, treatment-naïve status, year of ts/bDMARD initiation, concomitant methotrexate use, smoking status, and RF levels modeled as a continuous variable. Stabilized inverse probability weights were computed to estimate the average treatment effect in the overall population. Covariate balance before and after weighting was assessed using absolute standardized mean differences (SMDs) and visually examined using Love plots.

Changes in DAS28-ESR from baseline to 6 and 12 months were estimated using IPTW-weighted linear regression models. In addition, time to treatment discontinuation was assessed using IPTW-weighted Cox proportional hazards models, with robust variance estimators to account for the weighting and to obtain marginal hazard ratios. The proportional hazards assumption was formally assessed using Schoenfeld residuals. Patient-level cluster-robust standard errors were used to account for the non-independence of repeated treatment episodes contributed by the same patient.

All statistical tests were two-sided, and a p-value <0.05 was considered statistically significant. Data management and statistical analyses were performed using RStudio version 1.4.

### Handling of missing data

Patients with missing baseline RF levels were excluded from the study. Missing data were handled using multiple imputation by chained equations (MICE). Imputation was performed on a predefined set of baseline and follow-up variables considered clinically relevant. Continuous variables were imputed using predictive mean matching, binary variables using logistic regression, and categorical variables with more than two categories using multinomial logistic regression. To preserve the temporal structure of the data, future DAS28 values (6 and 12 months) were not used to impute baseline DAS28, while baseline DAS28 was allowed to inform the imputation of follow-up values. Twenty imputed datasets were generated with 20 iterations each, and convergence was assessed visually using trace plots and distributional diagnostics. The percentages of patients with missing data for each variable are shown in Supplementary Table S1.

Given the possibility of informative missingness in DAS28-ESR at 12 months, an additional sensitivity analysis was performed under a missing-not-at-random (MNAR) assumption. Among patients with missing 12-month DAS28-ESR who discontinued treatment for inefficacy before the 12-month assessment, no improvement from baseline was conservatively assumed by assigning the baseline DAS28-ESR value at 12 months.

All patients had available data on the date of treatment initiation or withdrawal.

## Results

### Description of the population

A total of 377 patients who underwent 417 treatments were initially included. However, 7 patients were excluded due to missing baseline RF data. Thus, the final dataset consisted of 370 patients who received a total of 409 treatments: 234 (57.2%) CZP and 175 (42.8%) with JAKi (66 baricitinib, 70 filgotinib, 19 tofacitinib and 20 upadacitinib).

A total of 81.9% of the patients were female, with a mean age of 52.0 years (SD 12.3) and a mean disease duration of 10.9 years (SD 9.5) (Table 1). Half of the patients (47.9%) were naïve to ts/bDMARDs, and 46.0% were receiving concomitant methotrexate.

**Table 1:** Baseline characteristics of the total population and stratified by treatment group.

Variable	Total *N* = 409	CZP *N* = 234	JAKi *N* = 175	*P*-value
Age, mean (SD)	52.0 (12.3)	50.9 (13.2)	53.6 (10.8)	0.026
Sex (female), *n* (%)	335 (81.9%)	190 (81.2%)	145 (82.6%)	0.763
Smoker, *n* (%)	137 (33.5%)	58 (24.8%)	79 (45.1%)	<0.001
Disease duration^a^, mean (SD)	10.9 (9.5)	8.5 (7.4)	14.1 (10.9)	<0.001
Concomitant MTX, *n* (%)	188 (46.0%)	104 (44.4%)	84 (48.0%)	0.479
ACPA positive, *n* (%)	293/370 (79.2%)	153/202 (75.7%)	140/168 (83.3%)	0.096
RF positive, *n* (%)	302 (73.8%)	166 (70.9%)	136 (77.7%)	0.153
Naïve to ts/bDMARD, *n* (%)	195 (47.9%)	126 (54.3%)	69 (39.4%)	0.005
DAS28-ESR, mean (SD)	4.6 (1.3)	4.6 (1.4)	4.5 (1.2)	0.520

ACPA, anti-citrullinated protein antibodies; CZP, certolizumab pegol; DAS28-ESR, disease activity score using 28 joint counts; JAKi, JAK inhibitors (baricitinib, filgotinib, tofacitinib and upadacitinib); MTX, Methotrexate; RF, rheumatoid factor; ts/bDMARD, targeted synthetic or biologic disease-modifying antirheumatic drug; SD, standard deviation.

a Disease duration refers to the time between the onset of symptoms and the initiation of the treatment evaluated in this study.

Baseline differences between the CZP and JAKi groups are shown in Table 1. Patients treated with JAKi had a longer disease duration (14.1 [10.9] vs. 8.5 [7.4] years, p < 0.001) and they were less frequently naïve to ts/bDMARDs (39.4% vs. 54.3%, p = 0.005). However, treatment with concomitant methotrexate was similar between groups (48.0% vs. 44.4% for JAKi and CZP groups, respectively, p = 0.479). Notably, smoking was more common in the JAKi group (45.1% vs. 24.8%, p < 0.001). Sex distribution, ACPA positivity, RF positivity, and disease activity measured by DAS28-ESR were similar between groups.

### Clinical effectiveness of CZP versus JAKi according to baseline RF level

**Figure 1:**
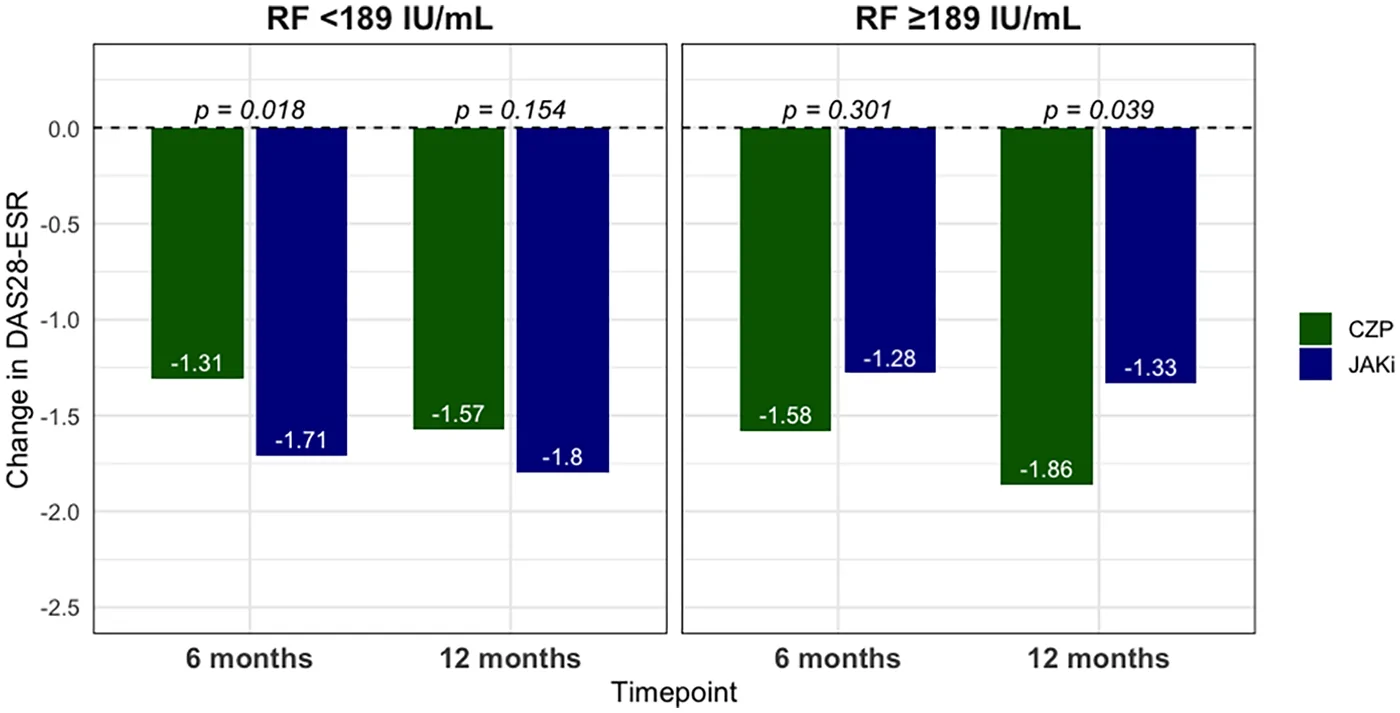
Change in DAS28-ESR from baseline to 6 and 12 months in patients with RF ≥189 IU/mL and <189 IU/mL, treated with CZP or JAK inhibitors. Error bars represent standard deviation. *P*-values indicate differences between treatments at each time point. CZP, certolizumab pegol; JAKi, JAK inhibitors; RF, rheumatoid factor.

In the overall population, both CZP and JAKi showed a reduction in DAS28-ESR at 6 months (–1.38 [1.38] vs. −1.61 [1.55], *p* = 0.121) and 12 months (–1.64 [1.41] vs. −1.69 [1.37], *p* = 0.953), with no significant differences between treatments. However, among patients with RF levels ≥189 IU/mL, CZP showed a more pronounced decrease in DAS28-ESR compared to JAKi at both 6 months (–1.58 [1.36] vs. −1.28 [1.53], *p* = 0.301) and 12 months (–1.86 [1.31] vs. −1.33 [1.21], *p* = 0.039), reaching statistical significance at 12 months (Figure 1). In contrast, among patients with RF <189 IU/mL, reductions in DAS28-ESR were greater in the JAKi group at both 6 months (–1.31 [1.38] vs. −1.71 [1.55], *p* = 0.018) and 12 months (–1.57 [1.43] vs. −1.80 [1.40], *p* = 0.154), with the difference reaching statistical significance at 6 months.

### Treatment retention of CZP versus JAKi according to baseline RF level

**Figure 2:**
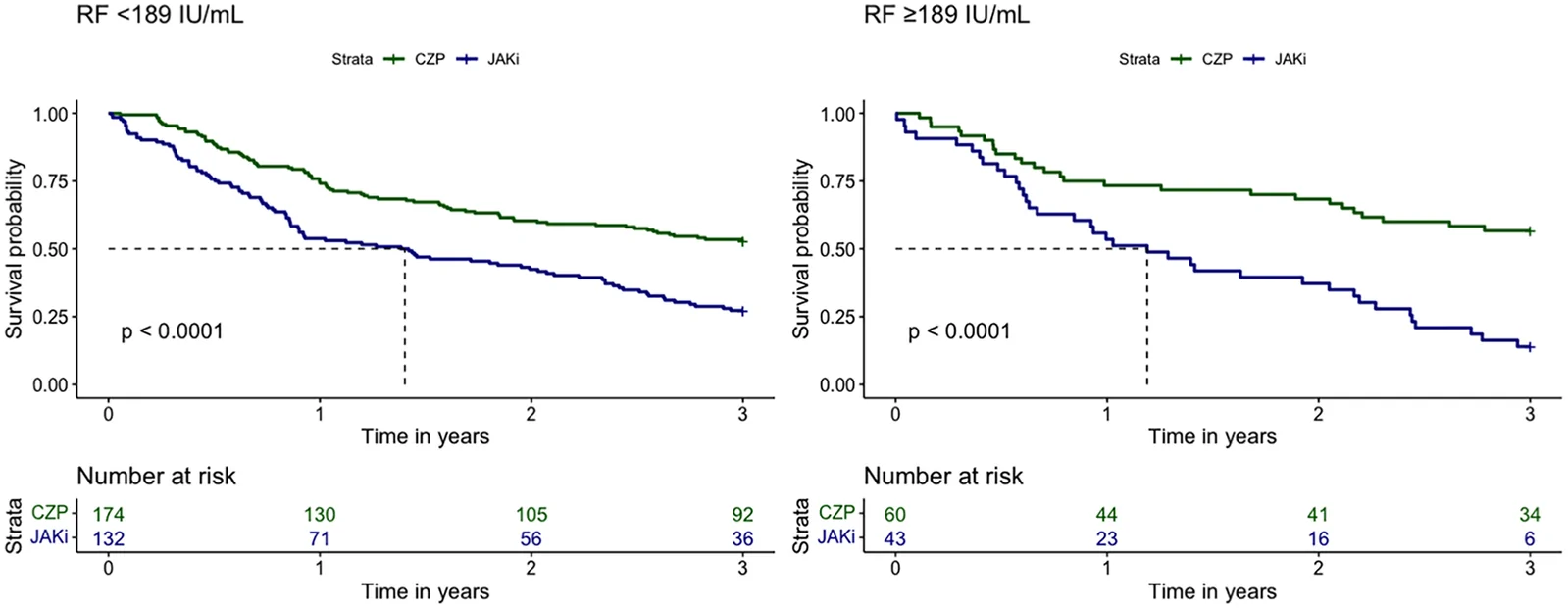
Treatment retention curves for CZP and JAKi stratified by rheumatoid factor level. **(Panel A)** RF <189 IU/mL. **(Panel B)** RF ≥189 IU/mL.

The retention rate of treatments after 3 years of follow-up was significantly higher for CZP compared to JAKi across both RF strata (Table 2 and Figure 2). In the overall population, the probability of drug survival at 3 years was 53.9% (95%CI: 47.8–60.6) for CZP and 24.0% (95%CI: 18.4–31.2) for JAKi, with a statistically significant difference (log-rank test, *p* < 0.001).

**Table 2:** Retention rates by baseline rheumatoid factor levels.

Population	Treatment	*N*	Number of withdrawals	Probability of drug survival after 3 years (95% CI)	*p*-value Log Rank test	HR (95% CI)	*p*-value HR
Overall population	CZP	234	108 (46.2%)	53.9 (47.8–60.6)	<0.001	Reference	<0.001
	JAKi	175	133 (76.0%)	24.0 (18.4–31.2)		2.2 (1.7–2.8)	
RF ≥189 IU/mL	CZP	60	26 (43.3%)	56.7 (45.4–70.7)	<0.001	Reference	<0.001
	JAKi	43	37 (86.0%)	13.9 (6.6–29.3)		2.9 (1.7–4.8)	
RF <189 IU/mL	CZP	174	82 (47.1%)	52.9 (45.9–60.8)	<0.001	Reference	<0.001
	JAKi	132	96 (72.7%)	27.3 (20.6–36.0)		2.0 (1.5–2.7)	

CI, confidence Interval; CZP, certolizumab pegol; HR, hazard ratio; JAKi, JAK inhibitors (baricitinib, filgotinib, tofacitinib and upadacitinib); RF, rheumatoid factor.

**Figure 3:**
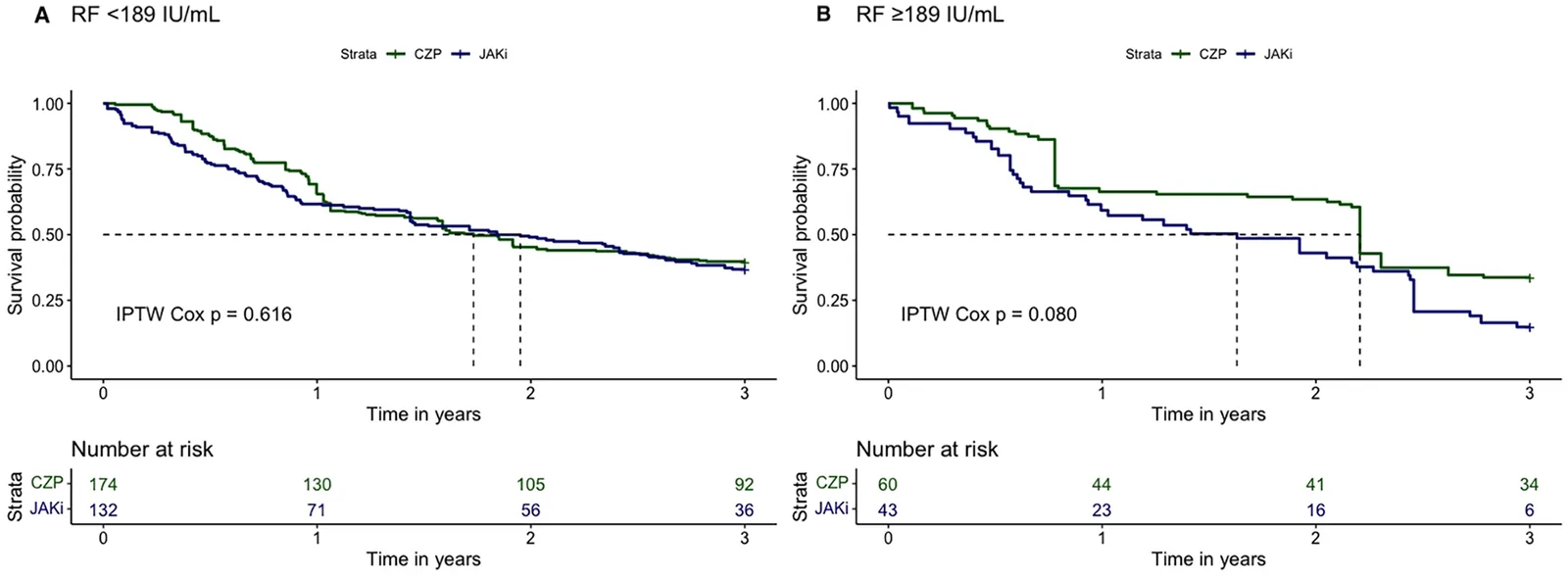
IPTW-adjusted treatment retention curves for CZP and JAKi stratified by rheumatoid factor level. **(Panel A)** RF <189 IU/mL. **(Panel B)** RF ≥189 IU/mL. Curves were estimated using inverse probability of treatment weighting (IPTW). Numbers at risk represent the unweighted number of treatment episodes remaining under follow-up. *P*-values are derived from IPTW-weighted Cox models with patient-level cluster-robust standard errors.

The HR for treatment discontinuation with JAKi compared to CZP was 2.2 (95%CI: 1.7–2.8, *p* < 0.001). The significantly higher probability of drug survival with CZP compared to JAKi was consistent across both the RF ≥189 IU/mL and RF <189 IU/mL subgroups, with a greater effect observed in the former (HR: 2.9, 95%CI: 1.7–4.8, *p* < 0.001) than in the latter (HR: 2.0, 95%CI: 1.5–2.7, *p* < 0.001) (Table 2 and Figure 2).

During the three-year follow-up, 108 of 234 CZP treatment episodes (46.2%) and 133 of 175 JAK inhibitor treatment episodes (76.0%) ended in active treatment withdrawal. The remaining 126 CZP episodes (53.8%) and 42 JAK inhibitor episodes (24.0%) were censored at the end of their available follow-up. Among patients treated with CZP, 25% of withdrawals were due to intolerance or adverse events, 26% to primary failure (i.e., initial non-response), 42% to secondary failure (i.e., initial clinical response followed by subsequent inefficacy), and 7% to other non-specified reasons. In the JAKi group, 40.5% of withdrawals were due to intolerance or adverse events, 19% to primary failure, 34.2% to secondary failure, and 6.3% to other non-specified reasons.

Among patients who discontinued treatment (Supplementary Figure S1), the timing of inefficacy-related discontinuation did not differ between CZP and JAK inhibitors in the RF <189 IU/mL stratum (*p* = 0.690), whereas a trend towards earlier discontinuation with JAK inhibitors was observed in the RF ≥189 IU/mL stratum (*p* = 0.058). Adverse-event-related discontinuation occurred earlier with JAK inhibitors in the RF <189 IU/mL stratum (*p* = 0.015), while no statistically significant difference was observed in the RF ≥189 IU/mL stratum (*p* = 0.280).

### IPTW-weighted sensitivity analysis stratified by rheumatoid factor levels

Covariate balance improved after IPTW, although substantial residual imbalance remained for year of treatment initiation (Supplementary Figure S2). In sensitivity analyses using IPTW, treatment effects on the change in DAS28-ESR were consistent with the main results (Table 3). Among patients with RF <189 IU/mL, JAK inhibitors were associated with a greater reduction in DAS28-ESR at 6 months compared with CZP (*β* = 0.52; 95% CI 0.08 to 0.97), with a similar but non-significant trend at 12 months. In contrast, among patients with RF ≥189 IU/mL, CZP was associated with a greater reduction in DAS28-ESR, reaching statistical significance at 12 months (*β* = −0.75; 95% CI −1.33 to −0.17).

**Table 3:** Treatment effects on the change in DAS28-ESR from baseline to 6 and 12 months estimated using weighted linear regression models.

Population	Visit	CZP vs. JAKi β (95%CI)	*p*-value
RF ≥189 IU/mL	6 months	−0.66 (−1.50 to 0.18)	0.120
	12 months	−0.75 (−1.33 to −0.17)	0.012
RF <189 IU/mL	6 months	+0.52 (0.08 to 0.97)	0.021
	12 months	+0.35 (−0.05 to 0.74)	0.088

CI, confidence interval; CZP, certolizumab pegol; HR, hazard ratio; JAKi, JAK inhibitors (baricitinib, filgotinib, tofacitinib and upadacitinib); RF, rheumatoid factor.

In an additional sensitivity analysis addressing potentially informative missingness, patients with missing 12-month DAS28-ESR who had discontinued treatment for inefficacy before the 12-month assessment were conservatively assumed to have experienced no improvement from baseline. In the high-RF subgroup, the direction of the treatment effect remained favourable to CZP, but the magnitude was attenuated and the difference was no longer statistically significant (*β* = −0.27, 95% CI −0.81 to 0.27; *p* = 0.316).

In sensitivity analyses using IPTW-weighted Cox proportional hazards models, no evidence of violation of the proportional hazards assumption was observed in either RF stratum (Supplementary Table S2). After IPTW adjustment, treatment retention was similar between CZP and JAKi among patients with RF <189 IU/mL (HR for discontinuation with JAKi vs. CZP 1.11, 95% CI 0.74–1.64; *p* = 0.616). Among patients with RF ≥189 IU/mL, JAKi treatment was associated with a numerically higher hazard of discontinuation than CZP (HR 1.65, 95% CI 0.94–2.91; *p* = 0.080) (Table 4). At 2 years, the IPTW-adjusted estimated treatment-retention probabilities were 63.4% with CZP and 43.0% with JAKi in the high-RF subgroup (Table 5).

**Table 4:** IPTW-adjusted hazard ratios for treatment discontinuation stratified by baseline rheumatoid factor levels.

Population	Treatment	HR (95% CI)	*p*-value HR
RF ≥189 IU/mL	CZP	Reference	0.080
	JAKi	1.6 (0.9–2.9)	
RF <189 IU/mL	CZP	Reference	0.616
	JAKi	1.1 (0.7–1.6)	

Hazard ratios (HRs) were estimated using IPTW-weighted Cox proportional hazards models with patient-level cluster-robust standard errors to account for repeated treatment episodes.

CI, confidence interval; CZP, certolizumab pegol; IPTW, inverse probability of treatment weighting; JAKi, JAK inhibitors (baricitinib, filgotinib, tofacitinib and upadacitinib); RF, Rheumatoid Factor.

**Table 5:** IPTW-adjusted treatment retention probabilities for CZP and JAKi according to rheumatoid factor level.

Population	Treatment	1-year retention, % (95% CI)	2-year retention, % (95% CI)	3-year retention, % (95% CI)
RF ≥189 IU/mL	CZP	66.3 (44.2–99.6)	63.4 (41.6–96.7)	33.7 (19.3–59.1)
	JAKi	59.3 (43.5–80.9)	43.0 (27.0–68.7)	14.9 (6.4–34.5)
RF <189 IU/mL	CZP	65.4 (55.9–76.6)	45.3 (36.5–56.2)	39.3 (31.2–49.6)
	JAKi	61.7 (51.8–73.4)	49.1 (38.7–62.1)	36.8 (26.6–51.0)

Treatment retention probabilities were estimated from IPTW-weighted survival curves. Values are presented as percentages with 95% confidence intervals.

CI, confidence interval; CZP, certolizumab pegol; IPTW, inverse probability of treatment weighting; JAKi, JAK inhibitors (baricitinib, filgotinib, tofacitinib and upadacitinib); RF, rheumatoid factor.

## Discussion

In this retrospective multicentre study, we investigated the effectiveness and retention rate of CZP versus JAKi according to baseline RF levels. Using an IPTW approach (balancing sex, age, disease duration, treatment-naïve status, year of ts/bDMARD initiation, concomitant methotrexate use, smoking status, and RF levels across groups), CZP was associated with better clinical response at 6 and 12 months compared to JAKi in patients with high RF levels (≥189 IU/mL), with the most pronounced between-group difference observed at 12 months. In the unadjusted analyses, CZP showed longer treatment retention than JAKi in both RF strata. After IPTW adjustment, treatment retention was similar between CZP and JAKi among patients with RF <189 IU/mL, whereas numerically higher treatment retention with CZP persisted among those with RF ≥189 IU/mL.

High levels of RF have been associated with higher disease activity (4, 13) and poorer clinical response to TNFi (14). Our findings are consistent with previous evidence suggesting that the effectiveness of CZP may be less influenced by RF levels (8). In contrast, patients with high RF treated with JAKi showed a smaller improvement in DAS28-ESR, despite having baseline disease activity comparable to that of patients treated with CZP. Nevertheless, the subgroup estimates had limited precision, and the lack of statistically significant differences at some timepoints should not be interpreted as evidence of equivalent effectiveness between treatments.

The good performance of CZP in patients with high RF levels may be biologically plausible due to its unique structure lacking an Fc region, which could prevent the formation of immune complexes commonly associated with Fc-containing bDMARDs (1). However, JAKi act via an intracellular mechanism and are theoretically unaffected by RF. Therefore, the reduced clinical response of JAKi in this patient profile is not readily explained from a biological standpoint.

It has been demonstrated that seropositive RA patients exhibit higher levels of inflammatory cytokines, including TNFα, compared to seronegative patients (15). One possible hypothesis is that a predominantly TNF-driven inflammatory profile in patients with high RF levels could favour a direct TNF-blocking strategy. Although JAKi inhibit the intracellular signalling pathways of several cytokines, differences in the underlying inflammatory phenotype could potentially influence the relative clinical response to these therapeutic mechanisms. This interpretation remains speculative and cannot be established from the present observational data. Another potential explanation is that patients with high RF may have a more aggressive disease phenotype, possibly contributing to a lower therapeutic response to JAKi. Additionally, one could assume that some patients may receive reduced doses of JAKi due to safety concerns, potentially leading to a poorer treatment response. However, only 6.7% of patients in this study were treated with half doses of JAKi, making dose reduction an unlikely explanation for the overall findings.

Comparisons between other TNFi, such as adalimumab, and JAKi have been conducted in clinical trials (16–19). However, none of these studies specifically focused on the high-RF population. To our knowledge, this is the first study to directly compare the effectiveness and retention of CZP versus JAKi in patients with high RF levels. Therefore, the present findings provide hypothesis-generating evidence regarding the potential value of RF levels when comparing these treatment strategies, but they are not sufficient to support definitive treatment recommendations. The differences observed in high-RF patients should be confirmed in adequately powered prospective studies, together with experimental investigations into whether RF or the associated inflammatory phenotype can modify the response to JAK inhibition.

Interestingly, among patients with RF <189 IU/mL, JAKi treatment was associated with a greater reduction in DAS28-ESR than CZP at 6 months in the IPTW adjusted model. Previous head-to-head trials have shown that some JAKi, particularly baricitinib and upadacitinib, may provide greater early improvements in disease activity than adalimumab, although this advantage has not been consistently demonstrated across all JAKi or clinical outcomes (16–19). However, evidence that RF level modifies the relative response to JAKi versus TNFi remains limited. Therefore, the greater 6-month response to JAKi observed in the low-RF stratum is a novel finding that requires confirmation and should not be interpreted as establishing low RF as a predictive biomarker of preferential JAKi response.

The long retention rate of CZP in patients with high RF levels, as well as its superiority over other TNFi, has been previously demonstrated by our group (9). However, no prior analyses have compared it directly with JAKi. In the unadjusted analyses, CZP showed significantly longer treatment retention than JAKi in both RF strata. After IPTW adjustment, treatment retention was similar between CZP and JAKi in the RF <189 IU/mL stratum, whereas numerically higher retention with CZP persisted in the RF ≥189 IU/mL stratum. Drug retention is, however, a composite real-world outcome influenced not only by effectiveness, but also by tolerability, safety concerns, patient preference, adherence, physician experience, treatment availability, and prescribing practices. It should therefore not be interpreted as a direct measure of pharmacological efficacy.

Several factors may account for this difference. One possible explanation is the greater effectiveness of CZP at 12 months in our study, which could contribute to its higher retention. Additionally, CZP has a longer track record, and physicians may feel more confident using it long-term, particularly in patients with comorbidities or a high disease burden. Conversely, growing safety concerns associated with JAKi (such as increased risks of cardiovascular events, malignancies, and infections) may prompt earlier discontinuation, especially in older or high-risk patients (20). Changes in regulatory safety recommendations during the study period may also have influenced treatment selection and discontinuation decisions.

Among patients treated with JAKi, 40.5% of withdrawals were attributed to adverse events or intolerance, compared with 25% among those treated with CZP. Nevertheless, the reason-specific survival analyses should be interpreted cautiously because of the smaller number of events within each RF stratum. In particular, the analysis of inefficacy-related discontinuation in the high-RF group suggested a difference favoring CZP but did not reach the conventional threshold for statistical significance. An intriguing finding is the high prevalence of smoking among JAKi patients. This can be explained, first, by the prescription of JAKi in some patients before October 2022, when the Pharmacovigilance Risk Assessment Committee (PRAC) of the European Medicines Agency recommended measures to minimize the risk of serious side effects associated with these drugs (21), as well as the potential absence of suitable therapeutic alternatives in some patients. Although smoking status was included in the IPTW model, residual confounding related to cumulative tobacco exposure, changes in smoking behaviour, or incompletely recorded smoking history cannot be excluded.

Treatment adherence may also differ between the two strategies, although the direction of this effect is uncertain: some patients may prefer the less frequent subcutaneous administration of CZP, whereas others may favour the oral administration of JAKi. Furthermore, CZP and JAKi entered clinical practice at different times and were prescribed within different regulatory and therapeutic contexts. The year of treatment initiation was included in the IPTW model, and follow-up was restricted to three years, but these measures cannot eliminate secular-trend bias. Residual differences in physician familiarity, available alternatives, safety recommendations, and prescribing behaviour may therefore have contributed to the retention results. Moreover, because the two treatments were not equally available throughout the study period, complete treatment overlap across calendar years could not be achieved. Thus, although adjustment for year of treatment initiation and restriction of follow-up reduced temporal imbalance, residual confounding related to calendar time and changes in therapeutic practice cannot be fully excluded.

Taken together, the observed associations suggest that CZP may represent a relevant treatment option for patients with high RF levels. However, the present results should not be interpreted as demonstrating pharmacological superiority or as establishing that longer CZP retention necessarily translates into better long-term structural or functional outcomes. Such conclusions would require prospective comparative studies with detailed assessment of disease control, safety, adherence, patient preference, and radiographic progression.

Although this study did not directly assess cost-effectiveness, the longer retention of CZP could indirectly lead to long-term cost savings within healthcare systems. Higher drug retention reduces the need for frequent treatment switches, which are often associated with additional costs for new drug acquisition, monitoring, and managing potential adverse events or disease flares associated with less persistent therapies (22). Nevertheless, no conclusions regarding economic benefit can be drawn from the present data, as formal healthcare-resource use and cost-effectiveness outcomes were not evaluated.

One of the main strengths of this study is the use of an IPTW approach to address measured differences in baseline characteristics between treatment groups while retaining the full analytical cohort. It is well established that combining TNFi with methotrexate improves treatment outcomes, including greater efficacy, longer drug retention, and higher rates of remission or low disease activity (23, 24). Older patients frequently present with multiple comorbidities, which can lead to early treatment discontinuation. Moreover, administering TNFi as a second- or third-line therapy reduces the likelihood of achieving responses comparable to those seen in biologic-naïve patients (25). For these reasons, concomitant methotrexate use, age, treatment-naïve status, disease duration, smoking, and year of treatment initiation, among other variables, were included in the weighting model.

Another strength is the multicentre nature of the registry, which includes patients from different geographical areas and multiple treating rheumatologists, thereby increasing the representativeness of routine clinical practice. Multiple imputation was also used to reduce the loss of information associated with missing baseline and follow-up variables. Finally, the use of survival analysis to evaluate retention rates with follow-up restricted to three years reduced, although did not eliminate, the potential bias arising from the different periods of availability of JAKi and CZP. By considering patients still on treatment at the time of data collection as censored, the analysis avoids penalizing these ongoing observations.

This study also has limitations. Its retrospective and non-randomised design introduces the potential for selection bias, missing data, and residual confounding. Patients with missing baseline RF values or unavailable treatment initiation or withdrawal dates were excluded. Although IPTW adjusted for several clinically relevant baseline covariates, this method can only account for measured and adequately recorded variables. It cannot eliminate confounding due to unmeasured, unavailable, time-varying, or imperfectly measured factors.

More than 70% of the information on concomitant corticosteroid use was missing, precluding inclusion of this variable in the models. Information on comorbidities such as dyslipidemia, hypertension, or diabetes, as well as methotrexate dosage, was not available in this dataset, despite their potential influence on treatment selection, response, and discontinuation. Differences in disease severity, previous treatment failures, patient preference, adherence, physician prescribing behaviour, and reasons for choosing one treatment over another may therefore remain insufficiently captured. Missing DAS28-ESR values were more frequent at 12 months than at 6 months and were particularly common in the JAKi group. Although multiple imputation was performed using clinically relevant baseline and longitudinal information, the validity of this approach depends on assumptions regarding the missing-data mechanism. Therefore, the 12-month effectiveness estimates should be interpreted with particular caution.

The Clinical Disease Activity Index (CDAI) would have been preferable to DAS28-ESR, as it is less affected by IL-6 inhibition; however, this measure was not consistently available in the clinical records. The JAKi group included four different drugs with varying mechanisms and potencies, and the pooled analysis may obscure drug-specific effects. Subgroup analyses by agent were not feasible due to the small sample size for each drug. In addition, the potential modifying effect of previous biologic exposure on the association between RF levels and treatment effectiveness was not specifically investigated. Previous evidence suggests that the relationship between RF and CZP effectiveness may differ between biologic-naïve and biologic-experienced patients (26). Although previous biologic exposure was accounted for in the propensity score model, the sample size did not allow sufficiently precise subgroup or interaction analyses according to biologic-naïve versus biologic-experienced status. Therefore, potential effect modification by previous biologic exposure cannot be excluded and should be explored in larger studies.

Finally, this study was based on all eligible patients with available data from the multicentre registry; therefore, the sample size was determined by real-world data availability rather than by a priori power calculation. The high-RF stratum and the reason-specific survival analyses included relatively small numbers of patients and events, resulting in wide confidence intervals and limited precision. Accordingly, the absence of statistical significance, particularly in the low-RF stratum or at individual timepoints, should not be interpreted as evidence of equivalence between treatments, as type II error remains possible. Conversely, because several subgroups and timepoint comparisons were performed, statistically significant findings should also be interpreted cautiously and require external confirmation.

In summary, this multicentre retrospective study in RA patients suggests that CZP is associated with greater improvement in disease activity and numerically higher treatment retention among patients with high RF levels. However, given the observational design, these findings should be considered hypothesis-generating. Prospective comparative studies are needed to determine whether RF levels can contribute to treatment stratification and to clarify the biological mechanisms potentially underlying differences in response across therapeutic classes.

## Data Availability

The raw data supporting the conclusions of this article will be made available by the authors, without undue reservation.
