# Supplementary material for: Effectiveness and retention of certolizumab pegol and JAK inhibitors in rheumatoid arthritis: a multicenter retrospective study stratified by rheumatoid factor status
Source: Front Med (Lausanne). 2026 Sep 9;13:1865270. doi: 10.3389/fmed.2026.1865270 (PMC13597368; doi:10.3389/fmed.2026.1865270)
Supplement: Supplementary file 1 [file Datasheet1.pdf]

Supplementary material

Supplementary Table 1. percentages of patients with missing data for each variable.

Variable	N total	N missing	% missing	% missing CZP group	% missing JAKi group
**Age**	409	0	0%	0 (0%)	0 (0%)
**Sex (female)**	409	0	0%	0 (0%)	0 (0%)
**Smoker**	409	42	10.3%	37/234 (15.8%)	5/175 (2.9%)
**Disease duration***	409	60	14.7%	31/234 (13.2%)	29/175 (16.6%)
**Concomitant MTX**	409	2	0.5%	2/234 (0.9%)	0/175 (0%)
**Baseline RF**	409	0	0%	0 (0%)	0 (0%)
**ACPA positive**	409	39	7.8%	32/234 (13.7%)	7/175 (4%)
**Year of bDMARD**	409	0	0%	9 (0%)	0 (0%)
**Naïve to ts/bDMARD**	409	2	0.5%	2/234 (0.9%)	0 (0%)
**DAS28-ESR baseline**	409	27	6.6%	24/234 (10.3%)	2/175 (1.1%)
**DAS28-ESR month 6**	409	92	22.5%	48/234 (20.5%)	44/175 (25.1%)
**DAS28-ESR month 12**	409	146	35.7%	62/234 (26.5%)	84/175 (48%)

**Disease duration refers to the time between the onset of symptoms and the initiation of the treatment evaluated in this study. ACPA: anti-citrullinated protein antibodies; CZP: certolizumab pegol; DAS28-ESR: Disease Activity Score using 28 joint counts; JAKi: JAK inhibitors (baricitinib, filgotinib, tofacitinib and upadacitinib); MTX: Methotrexate; RF: rheumatoid factor; ts/bDMARD: targeted synthetic or biologic disease-modifying antirheumatic drug; SD: standard deviation.*

Supplementary Table 2. Assessment of the proportional hazards assumption in the IPTW-weighted Cox models.

	Schoenfeld test χ^2	p-value	PH assumption*
**RF ≥189 IU/mL**	0.014	0.910	Not violated
**RF <189 IU/mL**	2.590	0.110	Not violated

PH: Proportional Hazards; RF: Rheumatoid Factor.
**The proportional hazards assumption was assessed using scaled Schoenfeld residuals. A p-value <0.05 was considered evidence against the proportional hazards (PH) assumption.*

Supplementary Figure 1. Kaplan–Meier plots showing retention rates of CZP versus JAK inhibitors according to rheumatoid factor levels and reason for discontinuation.

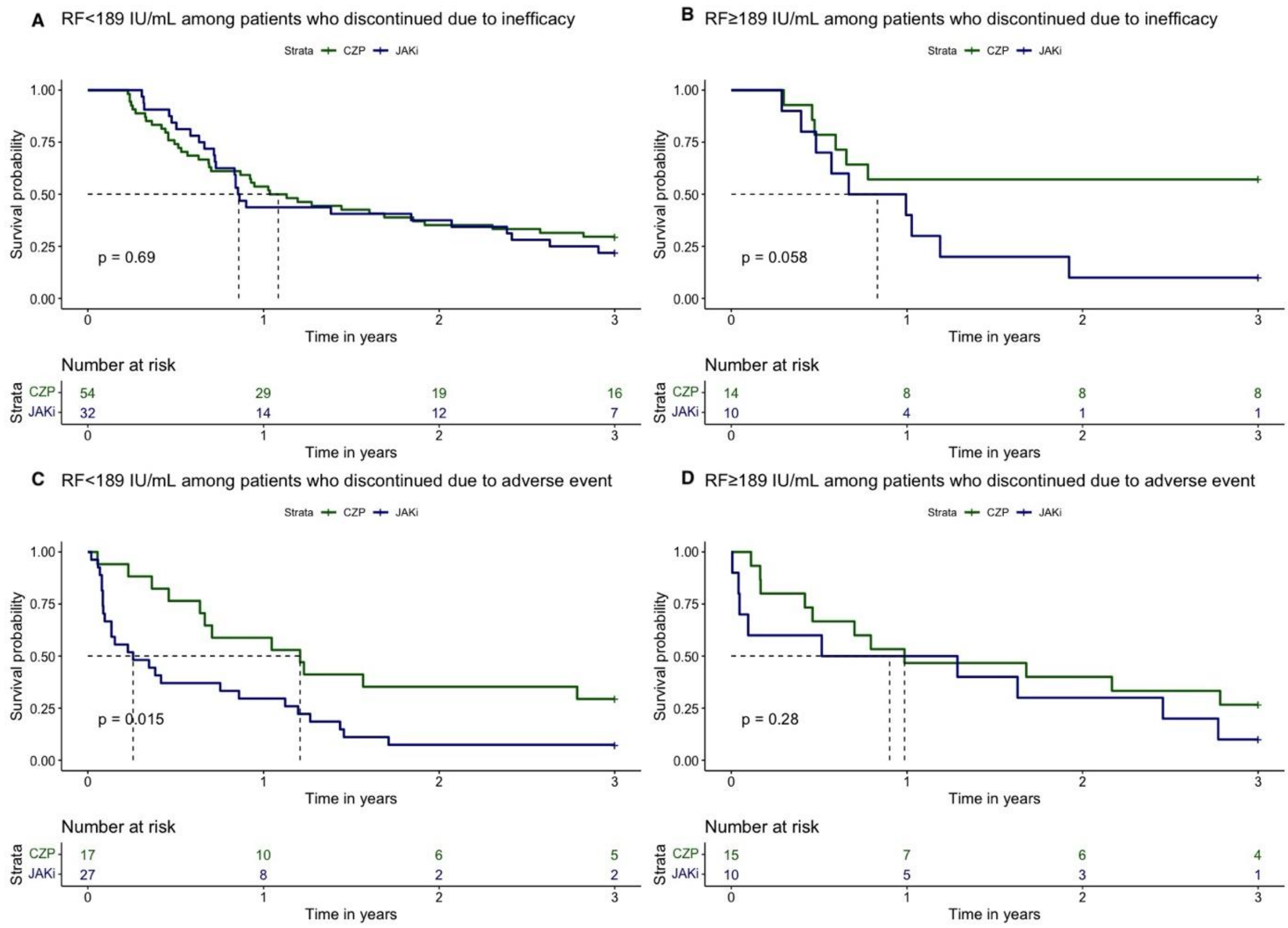

A)RF <189 IU/mL and discontinuation due to inefficacy; B) RF ≥189 IU/mL and discontinuation due to inefficacy; C) RF <189 IU/mL and discontinuation due to adverse events; and D) RF ≥189 IU/mL and discontinuation due to adverse events.

Supplementary Figure 2. Love plots showing covariate balance before and after inverse probability of treatment weighting, stratified by rheumatoid factor level.

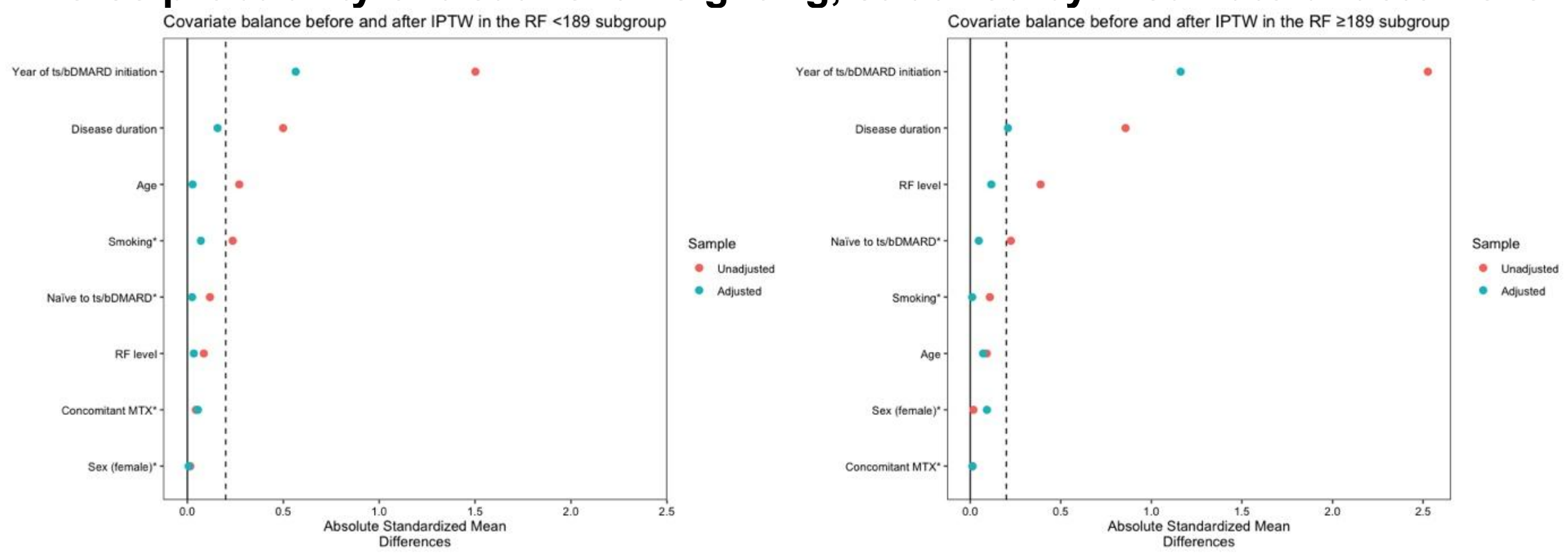

Legend: Love plots show the absolute standardized mean differences (SMDs) for baseline covariates before (unadjusted) and after IPTW adjustment in patients with (A) RF <189 IU/mL and (B) RF ≥189 IU/mL. The dashed vertical line indicates an absolute SMD of 0.20.
